# Automatic Outbreak Detection Algorithm versus Electronic Reporting System

**DOI:** 10.3201/eid1410.071354

**Published:** 2008-10

**Authors:** Masja Straetemans, Doris Altmann, Tim Eckmanns, Gérard Krause

**Affiliations:** Robert Koch Institute, Berlin, Germany; 1 Current affiliation: KNCV Tuberculosis Foundation, The Hague, the Netherlands

**Keywords:** infectious disease reporting, communicable disease control, algorithms, outbreaks, campylobacter, norovirus, surveillance, automatic data processing, signal detection, emerging, dispatch

## Abstract

To determine efficacy of automatic outbreak detection algorithms (AODAs), we analyzed 3,582 AODA signals and 4,427 reports of outbreaks caused by *Campylobacter* spp. or norovirus during 2005–2006 in Germany. Local health departments reported local outbreaks with higher sensitivity and positive predictive value than did AODAs.

In 2001, the Robert Koch Institute, Germany’s federal institute for infectious disease control, implemented an electronic system (SurvNet) for notifiable infectious disease surveillance (*1*,*2*). Local health departments electronically sent reports of confirmed cases to state health departments, which forwarded them to Robert Koch Institute. SurvNet can link single case reports to outbreak reports in which local health departments report descriptive outbreak information in a standardized manner (reported outbreaks). Additionally, the same software organizes the electronic transmission of single case reports from peripheral databases from each local health department to databases of the respective state health department and finally to Robert Koch Institute. Automatic outbreak detection algorithms (AODAs), run weekly on this case-based data, generate signals when the observed number of cases per a specific week is higher than a defined threshold value (signal outbreaks).

To identify the need to follow up generated signals, one must know the positive predictive value of AODA. This knowledge could avoid overwork in local health departments because not every signal will require contacting the local office for investigation.

Our goal was to assess the probability that a signal generated by AODA reflects a real outbreak (*Campylobacter* spp. or norovirus) being reported by local health department. Previous studies have tested AODAs by comparing generated signals with simulated outbreaks superimposed on authentic syndromic surveillance data (*3*,*4*) or with a limited number of known natural outbreaks (*5*). In contrast to these approaches, we evaluated performance of AODA by comparing it with a large database of outbreaks electronically reported by local health departments, which we considered to be the reference standard (*2*).

## The Study

We considered a signal outbreak to be identical to a reported outbreak when 1) >1 signal was triggered within the same period as the first and last case belonging to the particular reported outbreak, 2) the signal outbreak was associated with the identical geographic location on the municipal level (1 of the 430 municipalities) as the reported outbreak, and 3) the signal outbreak was associated with the identical pathogen (either *Campylobacter* spp. or norovirus). Using the data available as of June 1, 2007, we considered the number of reported outbreaks (a minimum of 4 cases because the algorithm cannot detect outbreaks with <4 cases), from week 5 of 2005 through week 4 of 2007.

**Figure 1:**
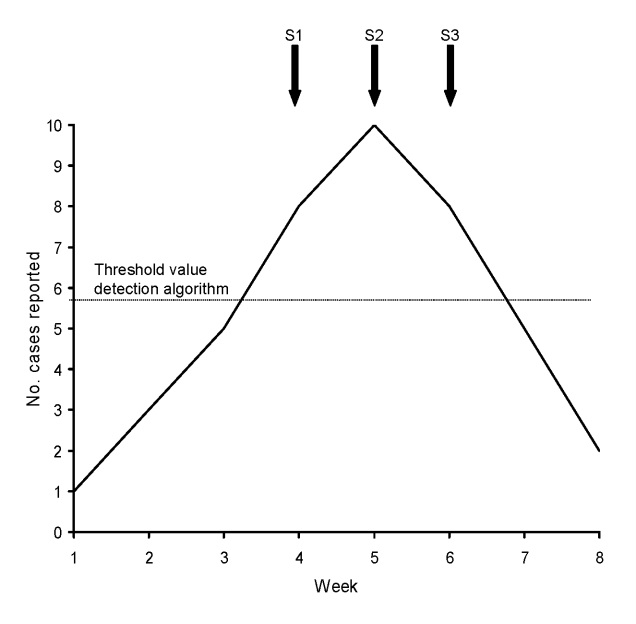
Example of 1 reported outbreak being detected by 3 signals. In this example, 3 signal outbreaks (S1, S2, S3) can be associated with 1 reported outbreak in same municipality and during the same period.

**Figure 2:**
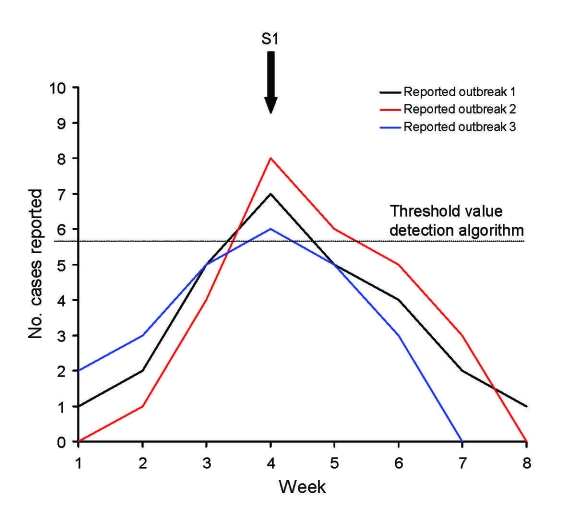
Example of 1 signal outbreak corresponding to multiple reported outbreaks. In this example, 1 signal outbreak (S1) can be associated with 3 reported outbreaks occuring in same municipality; threshold is reached in same week number.

During the study period, 118 and 4,309 outbreaks with >4 cases, associated with the pathogens *Campylobacter* spp. and norovirus, respectively, had been reported. The AODA had signaled 52 (44.1%) of the 118 reported *Campylobacter* spp. outbreaks and 2,538 (58.9%) of the 4,309 reported norovirus outbreaks (Table). The probability that a signal outbreak reflected a reported outbreak (positive predictive value of AODA) was lower for *Campylobacter* spp. than for norovirus: 50 (6.4%) of 781 *Campylobacter* spp. signal outbreaks and 2,115 (75.5%) of 2,801 norovirus signal outbreaks were associated with reported outbreaks. The AODA may have triggered multiple signals during the outbreak if the threshold level was reached during several consecutive weeks (Figure 1). Of the *Campylobacter* spp. outbreaks, 3 (6.0%) were each identified by 2 different signals; of the norovirus outbreaks, 727 (28.6%) were identified by multiple signals (2–20 signals per reported outbreak) (Table). Furthermore, 1 signal outbreak could correspond with different reported outbreaks when these occurred in the same local area and during the same period (Figure 2). For *Campylobacter* spp., 4 (8.0%) of the outbreak signals could correspond to >1 reported outbreak; for norovirus, 760 (35.9%) of the signal outbreaks could correspond to 2–26 reported outbreaks (Table).

**Table:** Outbreaks January 31, 2005–January 28, 2007, reported and identified by detection algorithm*

Outbreak characteristic	*Campylobacter* spp., no. (%)	Norovirus, no. (%)
Total cases	114,176	144,568
Cases as part of a reported outbreak	3,767 (3.3)	103,177 (71.4)
Reported outbreaks with <4 cases	1,453	5,074
Reported outbreaks with >4 cases	118	4,309†
Signal outbreaks generated by detection algorithm	781	2,801
Reported outbreaks with >4 cases identified by detection algorithm signals	52 (100)	2,538 (100)
Reported outbreaks identified by 1 signal	49 (94.0)	1,811 (71.4)
Reported outbreaks identified by >1 signal	3 (6.0)	727 (28.6)
Reported outbreaks identified by 2 signals‡	3 (6.0)	473 (18.6)
Reported outbreaks identified by >2 signals‡	0	254 (10.0)
Signal outbreaks corresponding to reported outbreak with >4 cases	50 (100)	2,115 (100)
Signal outbreaks corresponding to 1 reported outbreak	46 (92.0)	1,355 (64.1)
Signal outbreaks corresponding to >1 reported outbreak	4 (8.0)	760 (35.9)
Signal outbreaks corresponding to 2 reported outbreaks§	3 (6.0)	408 (19.3)
Signal outbreaks corresponding to >2 reported outbreaks§	1 (2.0)	352 (16.7)

*Data through June 1, 2007. Sensitivity detection algorithm 44.1% (52/118) for *Campylobacter* spp., 58.9% (2,538/4,309) for norovirus; no. reported outbreaks with >4 cases also identified by detection algorithm signal/total no. reported outbreaks with >4 cases. Positive predictive value of detection algorithm 6.4% (50/781) for *Campylobacter* spp.*,* 75.5 (2,115/2,801) for norovirus. No. signal outbreaks identical to reported outbreak/total number of signal outbreaks. **†**Excluded are 17 reported norovirus outbreaks of >25 wk and an average of <2 cases/wk because these are likely the result of data entry errors in SurvNet. ‡During the duration of a reported outbreak, the detection algorithm may have triggered multiple signals during several consecutive weeks (Figure 1). §One signal outbreak may correspond to multiple reported outbreaks if different outbreaks occur in the same municipality during the same period (Figure 2).

## Conclusions

Germany´s electronic reporting system for infectious disease outbreaks provided a unique opportunity to compare the triggering of signals through AODA with the reporting of outbreaks identified by local health departments. The probability of an outbreak signal being associated with a reported outbreak was much lower for *Campylobacter* spp. (6.4%) than for norovirus (75.5%). Furthermore, the fraction of cases as part of a reported outbreak was much lower for *Campylobacter* spp. (3.3%) than for norovirus (71.4%). Differences in route of transmission likely explain why *Campylobacter* spp. cases are generally more likely to occur sporadically and why norovirus cases are more likely to be part of an outbreak (*6*–*9*). These differences might result in a lower frequency of *Campylobacter* spp. outbreaks. The AODA might generate a signal when a higher than expected number of single cases is observed in a specific period and location, but this signal is likely to reflect an increased number of sporadic cases; an increased number of norovirus cases is more likely to reflect an occurring norovirus outbreak. An alternative possibility is that local health departments are more inclined to identify, investigate, and report norovirus outbreaks than *Campylobacter* spp. outbreaks (*10*). These differences demonstrate the importance of designing AODA specifically for the pathogens under surveillance.

For our analyses we used reported outbreaks as the reference standard by which to evaluate the AODA. Although this outbreak reporting is probably incomplete, we believe that it more closely identifies the true number of outbreaks than does retrospectively identifying outbreaks (*11*) or simulating outbreaks (*3*,*4*). Thus, we believe it generates a better reference standard than that used in previous studies.

Our findings question the usefulness of the AODA because a large number of generated signals were not confirmed by the electronic outbreak reporting from local health departments. Our results suggest that AODAs are not useful for detecting outbreaks on a local level because the outbreaks are detected earlier and investigated by the local health department. AODAs might be more useful for detecting multicounty or even multistate outbreaks, which are more difficult to detect by a single local health department. The latter has been well demonstrated by AODA detection of various foodborne outbreaks in Germany (*12*,*13*). National surveillance should focus on the follow-up of signals that indicate potential multicounty or multistate outbreaks. We used the county level for the algorithm because we obtain the reported outbreaks on this level first and we wanted to compare both systems. Our standard algorithms run also on federal and state levels, but that was not the subject of this investigation. To enable local health departments to earlier discover multicounty outbreaks, a new version of SurvNet is being developed. This version will give local health departments the opportunity to include more information on the evidence and also the possibility of linking outbreaks from different counties (*2*). The Robert Koch Institute, along with the state health departments, will develop a standard operating procedure for how to communicate and follow up on signals generated by the AODA.

Our study suggests that the usefulness of AODA to detect local outbreaks is limited because local health departments generally detect local outbreaks earlier and in more detail than these algorithms. Investment in the development of user-friendly outbreak reporting tools for local health departments might therefore provide better information on outbreaks than extensive refinements of AODAs.
